# Endothelial Phosphatase VE-PTP Participates in Vasculogenic Mimicry by Preventing Autophagic Degradation of VE-Cadherin

**DOI:** 10.3389/fonc.2020.00018

**Published:** 2020-01-24

**Authors:** Daniel Delgado-Bellido, Concepción Bueno-Galera, Laura López-Jiménez, Angel Garcia-Diaz, F. Javier Oliver

**Affiliations:** Instituto de Parasitología y Biomedicina López Neyra, CSIC, CIBERONC, Granada, Spain

**Keywords:** vasculogenic mimicry (VM), VE-PTP, vascular endothelial receptor protein tyrosine phosphatase, VE-cadherin, melanoma, autophagy

## Abstract

Aberrant extra-vascular expression of VE-cadherin has been observed in metastasis associated with Vasculogenic Mimicry (VM); we have recently shown that in VM prone cells VE-cadherin is mainly in the form of phospho-VE-cadherin in Y658 allowing increased plasticity that potentiates VM development in malignant cells. In the current study, we present results to show that human malignant melanoma cells VM+, express the VE-cadherin phosphatase VE-PTP. VE-PTP forms a complex with VE-Cadherin and p120-catenin and the presence of this complex act as a safeguard to prevent VE-Cadherin protein degradation by autophagy. Indeed, VE-PTP silencing results in complete degradation of VE-cadherin with the features of autophagy. In summary, this study shows that VE-PTP is involved in VM formation and disruption of VE-PTP/VE-Cadherin/p120 complex results in enhanced autophagy in aggressive VM^+^ cells. Thus, we identify VE-PTP as a key player in VM development by regulating VE-cadherin protein degradation through autophagy.

## Introduction

The term vasculogenic mimicry (VM) describes the formation of perfusion pathways in tumors by highly invasive, genetically deregulated tumor cells: vasculogenic because they distribute plasma and may contain red blood cells and mimicry because the pathways are not blood vessels and merely mimic vascular function. While VM formation is a marker of highly invasive tumor phenotype, mechanisms by which these structures may contribute to adverse outcome are not well-understood. It has been proposed that VM formation may facilitate tumor perfusion and the physical connection between VM and blood vessels may also facilitate hematogeneous dissemination of tumor cells. There is a strong association between the histological detection of VM patterns in primary uveal and cutaneous melanomas and subsequent death from metastasis (1, 2), consistent with the *in vitro* observations that these patterns are generated exclusively by highly invasive tumor cells (3). ECs express various members of the cadherin superfamily, in particular, vascular endothelial (VE-) cadherin (VEC), which is the primary adhesion receptor of endothelial adherent junctions. Aberrant extra-vascular expression of VE-cadherin has been observed in specific cancer types associated with VM (4). VE-PTP (vascular endothelial protein tyrosine phosphatase) is an endothelial receptor-type phosphatase whose name was coined for its prevalence to bind to VE-cadherin (5). VE-PTP poise endothelial barrier through helping homotypic VE-cadherin to keep at minimum basal endothelial permeability (6). Knockdown of VE-PTP increases endothelial permeability and leukocyte extravasation (7). VE-PTP also counterbalances the effects of permeability-increasing mediators such as VEGF, which increase endothelial permeability and leukocyte trafficking, by dephosphorylating VE-cadherin at Tyr658 and Tyr685, leading to stabilization of VE-cadherin junctions (8, 9).

p120-catenin was initially described as an Src kinase substrate, and then as a component of the cadherin-catenin complex. p120-catenin promotes cadherin stability, lowering the complex's susceptibility to endocytosis, ubiquitination, and proteasomal destruction (10). Phosphatases such as SHP-1, SHP-2, DEP1, and RPTPμ act upon p120-catenin. The RPTPμ tyrosine phosphatase binds p120 in a manner independent of p120's central Armadillo domain (11).

While studies have focused on the connection between VE-PTP and VE-cadherin in ECs. No reports have determined the role of VE-PTP in VM. Recent reports show that phospho-VE cadherin is highly expressed in VM+ cells and facilitates their pseudo-endothelial behavior by favoring p120/kaiso-dependent gene regulation (12). In the current study, we elucidated a mechanism linking VE-PTP expression with the induction of VM in metastatic melanoma cells: VE-PTP is present in the VE-Cadherin/p120 complex and the absence of VEPTP in this complex leads to autophagy. These results place VE-PTP as a dynamic component of VM transformation of melanoma cells owing to its ability to retain/safeguard VE-cadherin from being degraded by autophagy in aggressive cells.

## Results and Discussion

### VE-PTP Expression Is Essential for VE-Cadherin Stability and to Form VM

**Figure 1:**
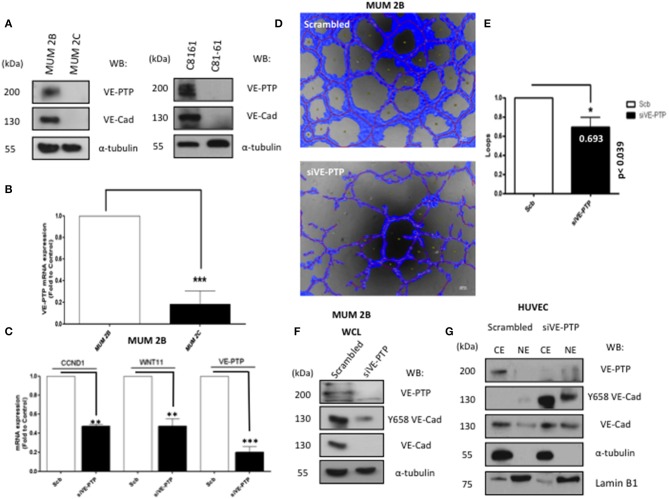
VE-PTP expression is essential for VE-cadherin stability and to form VM. **(A)** The expression of VE-PTP in aggressive (MUM 2B and C8161) and in non-metastatic (MUM 2C and C81-61) melanoma cells **(B)** RNA expression of VE-PTP MUM 2C cells **(C)** expression of kaiso-dependent genes (CCDN1 and WNT11) after silencing of VE-PTP decrease CCND1 and WNT 11 (Kaiso dependent genes) **(D,E)** siVE-PTP abolish the capacity to form *in vitro* VM in aggressive melanoma cells; images were acquired using an Olympus CKX41 microscope (bars 500 μm, the formation of tube-like structures was then quantified by Wimasis program. Each treatment was performed in triplicate, and the experiment was independently repeated at least three times. Results represented as fold enrichment over input. Asterisks denote statistically significant differences in an unpaired *t*-test (*p* < 0.05, ^**^p < 0.01, ^***^p < 0.001), and error bars denote SD, **(F)** to confirm the implications of VE-PTP in VM, we performed a siVE-PTP, and we show almost total decrease of VE-cadherin and Y658 expression in MUM 2B, **(G)** siVE-PTP in HUVEC in cytosol-nucleus subfractionation experiments showing the phosphorylation of Y658 VE-Cadherin.

Aberrant extra-vascular expression of VE-cadherin has been observed in specific cancer types associated with VM, and it has previously been shown that most of the VE-cadherin present in VM+ melanoma cells is phosphorylated form in Y658 (12). The current study is focused on the role of the phosphatase VE-PTP, its interaction with non-endothelial VE-cadherin and its consequences in VM development. Total VE-cadherin and VE-PTP expression were measured in different melanoma cell lines from either cutaneous (C8161, C81-61) or uveal (MUM 2B, MUM 2C) origin as shown in Figure 1A (protein) and Figure 1B (mRNA). Recently, our group reported that human malignant melanoma cells have a constitutively high expression of pVE-cadherin at position Y658, pVE-cadherin Y658 is a target of focal adhesion kinase (FAK) and forms a complex with p120-catenin and the transcriptional repressor Kaiso in the nucleus (12). We have also shown that FAK inhibition enabled Kaiso to suppress the expression of its target genes and enhanced Kaiso recruitment to KBS-containing promoters (CCND1 and WNT 11). Silencing of VE-PTP induced a significant reduction of CCND1 and WNT 11 (Kaiso-dependent genes) (Figure 1C) and disrupted VM formation quantified by Wimasis program (Figures 1D,E) suggesting that VE-PTP was also involved in the intracellular dynamic of VE-cadherin resulting in the regulation of Kaiso-dependent genes. To evaluate the correlation between the levels of VE-PTP and VE-cadherin we performed a western blot after siVE-PTP in MUM 2B (Figure 1F) and found an almost complete vanishing of VE-cadherin and pVE-cadherin suggesting that VE-PTP was involved in VE-cadherin stability and was needed for pVE-cadherin to reach the nucleus, as indirectly suggested the results obtained in Figure 1C. Interestingly, a completely different situation was found in primary endothelial cells HUVEC where siVE-PTP leads to an accumulation of pVEC in both cytosolic and nuclear compartments (Figure 1G). These results suggested that VE-PTP in malignant melanoma cells was protecting pVEC from degradation.

### VE-Cadherin and VE-PTP Form a Complex With p120 Catenin in Melanoma Cells

**Figure 2:**
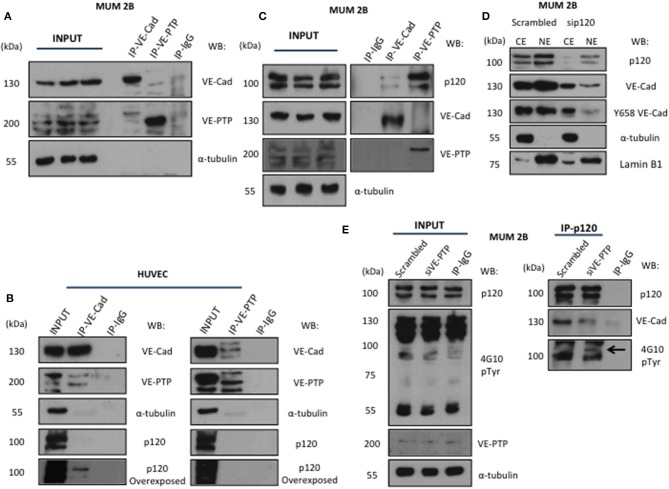
VE-Cadherin and VE-PTP form a complex with p120 catenin in melanoma cells: **(A)** immunoprecipitation of VE-Cadherin and VE-PTP in MUM 2B showed a little union of both proteins in VM cells, **(B)** in contrast, in endothelial cells (HUVEC) both proteins showed a robust interaction. **(C)** p120 interact with VE-PTP and VE-cadherin in immunoprecipitation experiments in MUM 2B **(D)** cytosol-nucleus subfractionation with scb and sip120 shown that the stability of VE-cadherin as well the phosphorylation of Y658 is p120-dependent, **(E)** p120 immunoprecipitation after VE-PTP silencing produce a high increase onto p120 phosphorylation and decrease the binding of VE-cadherin.

The VE-cadherin-catenin complex provides the backbone of the adherent junction in the endothelium. Nonetheless, in non-endothelial cells, the proteins interacting with VE-cadherin have not been identified. Using a coIP or co-immunofluorescence (Figure S1A) approach to analyse the VE-cadherin and VE-PTP interacting proteins showed that VE-Cadherin forms a fragile complex with VE-PTP in MUM2B (Figures 2A,C) compared with the stronger complex in HUVECs (used as a positive control on VE-PTP/VE-Cadherin complex in normal endothelial cells) (13) (Figure 2B). Surprisingly, the presence of p120-catenin in complex with VE-PTP appears in MUM2B cells as compared to HUVEC cells (Figures 2B,C) suggesting that VE-PTP might be involved in the control of p120-catenin phosphorylation status in melanoma cells. To analyse the possible impact of p120 on VE-cadherin stability in MUM2B, cytosol-nucleus subfractionation assay after silencing p120, found that p120 protect the stability of VE-cadherin (Figure 2D). Finally, we performed a coIP of p120 after siVE-PTP in MUM 2B cells, and we observed that binding of VE-cadherin to p120 was lost and resulted in increased global tyrosine phosphorylation of p120, suggesting that VE-PTP safeguard of VE-Cadherin/p120 binding in VM^+^ cells by balance between phosphorylation and dephosphorylation of p120 through VE-PTP activity (Figure 2E) and p120-catenin is likely to be a substrate for VE-PTP. These results are compatible with the increased phospho-p120 (as result of VE-PTP inactivation) being responsible for complex dissociation to initiate VE-cadherin proteolysis. In fact, it has been described that only isoform 1A of p120 can be substrate of RPTPmu, correlating with the results in Figure 2C (increased binding of VE-PTP to isoform 1A of p120) and 2E (see the band with the arrow corresponding to pY-p120 in the blot for global p-Try (4G10) in the immunoprecipitation of p120).

### VE-Cadherin/VE-PTP Complex Dissociation-Enhanced Autophagy

**Figure 3:**
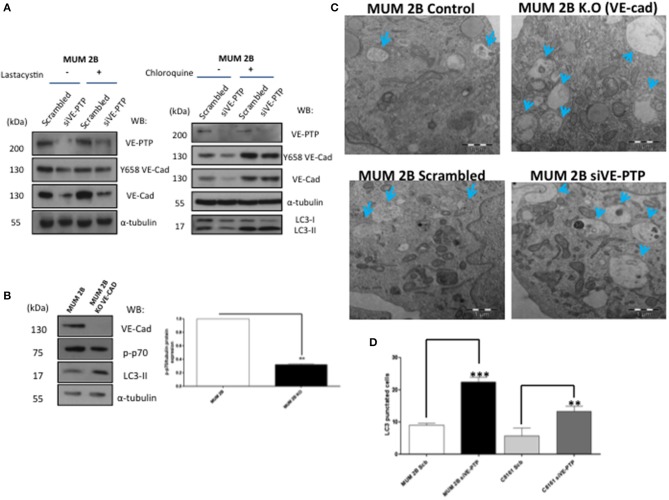
VE-Cad/VE-PTP complex dissociation-enhanced autophagy: **(A)** inhibition of proteasome with lactacystin (30 μM during 30 min) with or without siVE-PTP or inhibition of autophagy with chloroquine treatment (20 μM during 3 h) **(C)** electron microscopy experiments in control (scrambled), siVE-PTP and MUM 2B K.O; autophagosomes are shown with arrows (bars: 1μm), **(B)** Western blot of p-p70 and LC3-II **(D)** quantification of autophagosomes (LC3-GFP punctuated cells) following silencing of VE-PTP in both MUM 2B and C8161 VM^+^ cells, each treatment was performed in triplicate, and the experiment was independently repeated at least three times. Results represented as fold enrichment over input. Asterisks denote significance in an unpaired *t*-test (*p* < 0.05, ^**^p < 0.01, ^***^p < 0.001), and error bars denote SD.

To elucidate the mechanism leading to VE-Cadherin degradation after the inhibition of VE-PTP, we treated MUM2B cells with proteasome inhibitor lactacystin or MG-132 (Figure S1B) and did not prevent VE-Cadherin degradation after VE-PTP disabling (Figure 3A) suggesting a proteasome-indenpendent pathway. Macroautophagy (referred to only as “autophagy”) is a homeostatic “self-eating” pathway that has been conserved among eukaryotic cells. This is a lysosomal-associated process in which intracellular components, small portions of cytosol or chaperone-associated cargo, are engulfed in double-membrane vesicles, called autophagosomes, to be degraded with lysosomal hydrolyses (14). Recently, different studies reported that the formation of VM was promoted by bevacizumab-induced autophagy in GSCs, which was associated with tumor resistance to antiangiogenic therapy through the high expression of VEGFR-2 (15). In our study, inhibition of the fusion of autophagosomes and lysosomes with chloroquine suppressed the degradation of VE-cadherin after siVE-PTP (Figure 3A and Figure S1C). Even more, the levels of the mTOR substrate p-p70 (as a readout of mTOR activity and autophagy status) decreased in MUM 2B knockout for VE-cadherin (Figure 3B) suggesting that the complex VE-cadherin/VE-PTP might be restraining autophagy. Electron microscopy experiments (Figure 3C) were performed in MUM 2B and C8161 cell deficient for siVE-PTP or MUM 2B knockout for VE-cadherin; these results showed an enhanced autophagic morphology after the VE-PTP silencing or in VE-cadherin knockout cells, suggesting that the absence of either protein may have implications in the dynamic of protein turnover involoving the activation of autophagy. To confirm the implication of autophagy on VE-cadherin degradation, we quantified autophagosomes formation under the same conditions described above after transfection of LC3-GFP and observed that the number of autophagosomes (LC3-GFP punctuated) increased following silencing of VE-PTP in both MUM 2B and C8161 VM^+^ cells (Figure 3D and Figure S2C). By analyzing the cBioPortal database, a platform of 48333 tumor samples, we found that high mRNA levels of PTPRB (the gene encoding for VE-PTP) were inversely associated with the expression of two essential genes involved in autophagy, LAMP1 and ATG7 in uveal melanoma (Figure S2D), suggesting that in patients this interaction might be relevant to determine autophagic features of the tumor.

**Figure 4:**
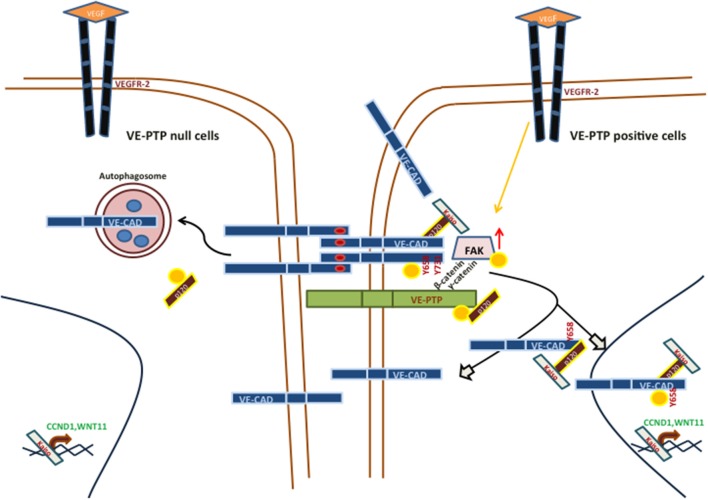
Main signaling pathways involved in vasculogenic mimicry in VE-PTP/VE-Cad null or positive, aggressive melanoma cells.

While the role of VE-cadherin as a determinant of the pseudo-endothelial behavior of malignant melanoma cells have been widely described, no studies have addressed so far the implications of VE-PTP in VM development. Previous results have reported that VE-cadherin in VM-prone tumor cells is mostly as pVE-cadherin (Y658) and in several intracellular locations (including the nucleus) conferring the cells with the necessary plasticity to undergo pseudo-endothelial differentiation (12). To get further information on the cause of these phosphorylated VE-cadherin population, we focalized in the phosphatase VE-PTP that keeps VE-cadherin unphosphorylated in endothelial cells. Despite the massive amounts of VE-cadherin, VE-PTP levels in melanoma cells were sharply diminished, suggesting that a majority of the VE-cadherin population is not in complex with VE-PTP (as compared with endothelial cells Figures 2A,B), then tolerating the accumulation of pVE-cadherin while endothelial cells tight junctions require a stable and abundant VE-PTP/VE-cadherin complex to keep vascular permeability strictly under control. In aggressive melanoma cells, that not presence (VE-PTP null cells) of unbound VE-Cadherin to VE-PTP initiates the proteolysis of VE-cadherin trough autophagy (increase p-p120) and finally decrease VM capacity or the reverse situation VE-PTP positive cells, increase the capacity to form VM (Figure 4). The question remains how a relatively small amount of VE-PTP protects from proteolysis and what signal emerges from the complex dissociation to activate autophagy. Contrary to endothelial cells, p120-catenin is also firmly attached to VE-PTP in MUM 2B cells (Figures 2B,C), and the loss of this complex (after siVE-PTP silencing) leads to p120-catenin increased phosphorylation and VE-cadherin degradation by disunity of p120. Globally these results shed light to a new mechanism to control VM through the balance between VE-PTP/VE-cadherin and the phosphorylation status of p120 in aggressive melanoma VM^+^ cells.

## Materials and Methods

### Reagents and Antibodies

The following reagents were used: Chloroquine 100 μM during 3 h, Lactacystin 30 μM during 30 min and MG-132 3 μM during 3 h. Corning Matrigel Basement Membrane Matrix for *in vitro* angiogenesis experiments. Antibodies used were: Y658 VEC rabbit (1:1000 WB, 1:100 IF, Thermofisher), VEC C-ter mouse (1:500 WB, 1:50 IF, 2μg IP, clone F-8, sc-9989), anti-phosphotyrosine p-Tyr mouse (1:1000 WB, clone 4G10, Millipore), α-tubulin mouse (1:10000 WB, clone B-5-1-2, Sigma-Aldrich), p120 catenin mouse (1:1000 WB, 1:100 IF, 2μg IP, BD Biosciences), lamin B1 rabbit (1:1000 WB, Abcam) and VE-PTP mouse (1:1000 WB,1:100 IF, 2μg IP, Clone 12/RPTPb, BD Biosciences).

### Cell Lines

Human melanoma cells MUM 2B, MUM 2C, C8161, and C81-61 were grown in RPMI medium supplemented with 10% fetal bovine serum, 2 mM of L-glutamine, and 1% penicillin/streptomycin (PAA laboratories). Human umbilical vein endothelial cells (HUVEC) were grown in endothelial cells growth medium-2 (EGM-2) (Lonza). All cells were cultured at 37°C and 5% CO2 in incubator cells.

### *In vitro* Angiogenesis Assay

The effect of siVE-PTP on the formation of tube-like structures in Matrigel (BD Biosciences) was determined according to the manufacturer's instructions. Briefly, 96-well plates were coated with 50 μl of BD Matrigel™ Basement Membrane Matrix and allowed to solidify at 37°C in 5% CO2 for 30 min. Cells were treated Scb, siVE-PTP transfected for 48h as described previously. After 48 h, respectively, of incubation, images were acquired using an Olympus CKX41 microscope (10X lens). The formation of tube-like structures quantified by Wimasis program. Each treatment was performed in triplicate, and the experiments independently repeated at least three times.

### Quantitative RT-PCR

Total RNA was isolated by RNeasy Mini Kit (Qiagen) according to the manufacturer's recommendations. About 1 μg of RNA from each sample was treated with DNase I, RNasinRibonuclease inhibitors (Invitrogen) and reverse-transcribed using iScriptcDNA synthesis kit (Biorad) following the manufacturer's protocols. cDNA was amplified using the iTaq Universal SYBR green supermix (Biorad). Each reaction was performed in triplicate using CFX96 Real-time PCR detection systems. Primer sequences for the targets and the annealing temperature (60°C): 36B4: Forward 5′-CAGATTGGCTACCCAACTGTT-3′, Reverse 5′-GGCCAGGACTCGTTTGTACC-3, CCND1: Forward 5′-CCGTCCATGCGGAAGATC-3′, Reverse 5′-GAAGACCTCCTCCTCGCACT-3′; WNT11: Forward 5′-GCTTGTGCTTTGCCTTCAC-3′, Reverse 5′-TGGCCCTGAAAGGTCAAGTCTGTA-3′, VE-PTP: Forward 5′-TGCTAAGTGGAAAATGGAGGCT-3′, Reverse 5′-GCCCACGACCACTTTCTCAT-3′.

### Gene Editing

MUM2B knockout (ko) cells for the VE-Cad gene were generated using the CRISPR-Cas9 technology. Five different sgRNAs were designed using the Zhang Lab Optimized CRISPR design tool and cloned into the pL-CRISPR.EFS.GFP which purchased from the Addgene public repository (#57818). sgRNA guides were validated in HEK293T Cells using the GeneArt Genomic Cleavage Detection Kit (Invitrogen, Carlsbad, USA) according to the manufacturer's instructions. Lentiviral particles for the best two sgRNAs in terms of allelic disruption (GGCAGGCGCCCGATGTGGCG and GATGATGCTCCTCGCCACATC).

### Transfection of Small Interfering siRNA

Cultured cells were transiently transfected with an irrelevant siRNA (5′-CCUACAUCCCGAUCGAUGAUG-3′) 50 nM. siVE-PTP: 5′- GACAGUAUGAGGUGGAAGU−3′, 50 nM, sip120: 5′- GGATCACAGTCACCTTCTA−3′, 50 nM, were transfected for 48 h using JetPrime (Polyplus transfection) according to the recommendations.

### Immunobloting, Immunoprecipitation, Subfractionation Cytosol-Nucleus

For simple coimmunoprecipitation, cells were lysed in lysis buffer (50 mM Tris/HCl ph 8, 120 mM NaCl, 0,1 % NP-40, 1 mM EDTA, 10 mM NaF, 1 mM Na3VO4 and supplemented with a protease inhibitor cocktail (1 tablets to 10 ml of lysis buffer, Roche) for 30 min at 4°C. Lysates were cleared by 13.000 rpm centrifugation for 10 min at 4°C and incubated overnight at 4°C with respective antibodies. Consequently, the next day, IP lysates were incubated for 2 h at 4°C with 50 μl of-of Dynabeads™ Protein G for Immunoprecipitation (ThermoFischer). Dynabeads were washed three times with low salt 120 mM lysis buffer and two times with high salt 300 mM lysis buffer. All lysates separated by dodecyl sulfate-polyacrylamide (7,5%, Biorad) gel electrophoresis and transferred to PVDF membrane (Pall laboratory) by semi-wet blotting.

Accordingly with the article of Rockstroh et al. (16), for subfractionation cytosol-nucleus, cells were lysed in lysis buffer (250 mM sucrose, 50 mM Tris-HCl ph 7,4, 5 mM MgCl2, 1 mM Na3VO4, 0,25 % NP-40 and supplemented with a protease inhibitor cocktail (1 tablet to 10 ml of lysis buffer, Roche) for 10 min at 4°C, lysates centrifuged at 500 g for 5 min, supernatant was considerate cytosolic fraction, pellet was resuspended in buffer 2 (1M sucrose, 50 mM Tris-HCl ph 7,4, 5 mM MgCl2) and centrifuged at 3,000 g for 5 min, supernatant was discarded. Pellet was resuspended in nuclear buffer (20 mM Tris-HCl ph 7,4, 0,4 M NaCl, 15% glycerol, 1,5% Triton X-100) for 45 min at 4°C in agitation. This lysate centrifuged at 5,000 g for 5 min; the supernatant was considered a nuclear fraction.

### Immunofluorescence

Immunostaining was performed on cells plated onto coverslips and grown for 24 h prior to experimental treatment. The cultured media was removed and wash two times with PBS 1X and the cells were fixed (Paraformaldehyde 3%, 5% sucrose) for 15 min at room temperature. Permeabilization was performed using 0,25% Triton-100 in PBS for 10 min. Before start with the antibodies incubation, cells were blocked with BSA 2% for 1 h. Respective primary antibodies were incubated for 45 min and secondary antibodies Alexa Fluor 488 anti-mouse (1:500, green) or Alexa Fluor 594 anti-rabbit (1:250, red) were incubated for 20 min. Nuclear counter staining with 4′,6′-diamidino-2-phenylindole dihydrochloride (DAPI) was performed after removal of secondary antibody. Immunofluorescence images were obtained in the linear range of detection to avoid signal saturation using a fluorescent microscoper confocal microscopy (Leica SP5, 63X lens).

### Electron Microscopy

The MUM 2B extracted were washed with PBS, prefixed for 30 min in a fixation solution (0.1 M cacodylate buffer pH 7.4 and osmium tetraoxide) for 60 min at 4°C. After this treatment, tissues were washed with MilliQ water, and the samples were stained with uranyl acetate. The ultrathin sections were cut with a diamond knife in an ultramicrotome (Reichert Ultracut S). The samples were analyzed in a TEM Zeiss 902 with 80 KV of voltage acceleration (CIC-UGR).

### Autophagy Assay

GFP-LC3-expressing cells have been used to demonstrate the induction of autophagy. The GFP-LC3 expression vector was kindly supplied by Dr T Yoshimori (National Institute for Basic Biology, Okazaki, Japan). MUM 2B and C8161 were transiently transfected (0,5 μgr) with this vector together with jetPrime (Polyplus transfection, Illkirch, France) according to the manufacturer's protocol. The assay was performed on cells grown in six-well plates. To determine LC3 localization, GFP-LC3-transfected cells were observed under a Zeiss (Zeiss Axio Imager A1) fluorescence microscope (20X lens). To determine LC3-II translocation, performed western blot of LC3-I and its proteolytic (phosphatidylethanolamine) derivative LC3-II (18 and 16 kDa, respectively) using a monoclonal antibody against LC3 (NanoTools,1:1000 WB, 1:100 IF, clone 5F10, Ref 03231-100/LC3-5F10).

## Data Availability Statement

All datasets generated for this study are included in the article/Supplementary Material.

## Author Contributions

All authors listed have made a substantial, direct and intellectual contribution to the work, and approved it for publication.

### Conflict of Interest

The authors declare that the research was conducted in the absence of any commercial or financial relationships that could be construed as a potential conflict of interest.

## Supplementary Material

Figure S1 **(A)** Co-immunofluorescence of VE-PTP (green) and VE-Cadherin (red), DAPI (nuclear stain, blue) in MUM 2B cells. Bars 15 μm. **(B)** Inhibition of proteasome through MG-132 (3 μM during 3 h) with or without siVE-PTP conditions not prevent the VE-cadherin degradation. **(C)** Co-immunofluorescence of Y658 VE-Cadherin (red) and LC3 I/II (green) with or without siVE-PTP (Cloroquine treatment: 20 μM during 3 h) conditions in MUM 2B cells. Bars 15 μm.

Figure S2 **(A)** Quantification of autophagosomes (LC3-GFP punctuated cells) in MUM 2B cells and C8161 cells after LC3-GFP transfection (0.5 μgr). **(B)** cBioPortal database, a platform of 48,333 tumors samples, we found that high mRNA levels of PTPRB, associated with the expression of two essential autophagy genes, ATG7 and LAMP1 in uveal melanoma samples.

## References

[1] McLean IW Keefe KS Burnier MN. Uveal melanoma. Comparison of the prognostic value of fibrovascular loops, mean of the ten largest nucleoli, cell type, and tumor size. Ophthalmology. (1997) 104:777–80. 10.1016/S0161-6420(97)30234-6 9160022

[2] Warso MA Maniotis AJ Chen X Majumdar D Patel MK Shilkaitis A. Prognostic significance of periodic acid-Schiff-positive patterns in primary cutaneous melanoma. Clin Cancer Res. (2001) 7:473–77. 11297236

[3] Maniotis AJ Folberg R Hess A Seftor EA Gardner LM Pe'er J. Vascular channel formation by human melanoma cells *in vivo* and *in vitro*: vasculogenic mimicry. Am J Pathol. (1999) 155:739–52. 10.1016/S0002-9440(10)65173-5 10487832 PMC1866899

[4] Hendrix MJ Seftor EA Meltzer PS Gardner LM Hess AR Kirschmann DA. Expression and functional significance of VE-cadherin in aggressive human melanoma cells: role in vasculogenic mimicry. Proc Natl Acad Sci USA. (2001) 98:8018–23. 10.1073/pnas.131209798 11416160 PMC35460

[5] Nawroth R Poell G Ranft A Kloep S Samulowitz U Fachinger G. VE-PTP and VE-cadherin ectodomains interact to facilitate regulation of phosphorylation and cell contacts. EMBO J. (2002) 21:4885–95. 10.1093/emboj/cdf497 12234928 PMC126293

[6] Wessel F Winderlich M Holm M Frye M Rivera-Galdos R Vockel M. Leukocyte extravasation and vascular permeability are each controlled in vivo by different tyrosine residues of VE-cadherin. Nat Immunol. (2014) 15:223–30. 10.1038/ni.2824 24487320

[7] Muller WA. How endothelial cells regulate transmigration of leukocytes in the inflammatory response. Am J Pathol. (2014) 184:886–96. 10.1016/j.ajpath.2013.12.033 24655376 PMC3969991

[8] Wallez Y Cand F Cruzalegui F Wernstedt C Souchelnytskyi S Vilgrain I. Src kinase phosphorylates vascular endothelial-cadherin in response to vascular endothelial growth factor: identification of tyrosine 685 as the unique target site. Oncogene. (2007) 26:1067–77. 10.1038/sj.onc.1209855 16909109

[9] Orsenigo F Giampietro C Ferrari A Corada M Galaup A Sigismund S. Phosphorylation of VE-cadherin is modulated by haemodynamic forces and contributes to the regulation of vascular permeability *in vivo*. Nat Commun. (2012) 3:1208. 10.1038/ncomms2199 23169049 PMC3514492

[10] Chiasson CM Wittich KB Vincent PA Faundez V Kowalczyk AP. p120-catenin inhibits VE-cadherin internalization through a Rho-independent mechanism. Mol Biol Cell. (2009) 20:1970–80. 10.1091/mbc.e08-07-0735 19211843 PMC2663933

[11] Zondag GC Reynolds AB Moolenaar WH. Receptor protein-tyrosine phosphatase RPTPmu binds to and dephosphorylates the catenin p120(ctn). J Biol Chem. (2000) 275:11264–9. 10.1074/jbc.275.15.11264 10753936

[12] Delgado-Bellido D Fernandez-Cortes M Rodriguez MI Serrano-Saenz S Carracedo A Garcia-Diaz A. VE-cadherin promotes vasculogenic mimicry by modulating kaiso-dependent gene expression. Cell Death Differ. (2018) 26:348. 10.1038/s41418-018-0125-4 29786069 PMC6329820

[13] Vockel M Vestweber D. How T cells trigger the dissociation of the endothelial receptor phosphatase VE-PTP from VE-cadherin. Blood. (2013) 122:2512–22. 10.1182/blood-2013-04-499228 23908467

[14] Dikic I Elazar Z. Mechanism and medical implications of mammalian autophagy. Nat Rev Mol Cell Biol. (2018) 19:349–64. 10.1038/s41580-018-0003-4 29618831

[15] Wu HB Yang S Weng HY Chen Q Zhao XL Fu WJ. Autophagy-induced KDR/VEGFR-2 activation promotes the formation of vasculogenic mimicry by glioma stem cells. Autophagy. (2017) 13:1528–42. 10.1080/15548627.2017.1336277 28812437 PMC5612353

[16] Rockstroh M Müller SA Jende C Kerzhner A Von Bergen M Tomm JM Cell fractionation - an important tool for compartment proteomics. J Integ OMICS. (2011) 1:135–43. 10.5584/jiomics.v1i1.52

