# Supplementary figures and images for: Endothelial Phosphatase VE-PTP Participates in Vasculogenic Mimicry by Preventing Autophagic Degradation of VE-Cadherin
Source: Front Oncol. 2020 Jan 24;10:18. doi: 10.3389/fonc.2020.00018 (PMC7025541; doi:10.3389/fonc.2020.00018)

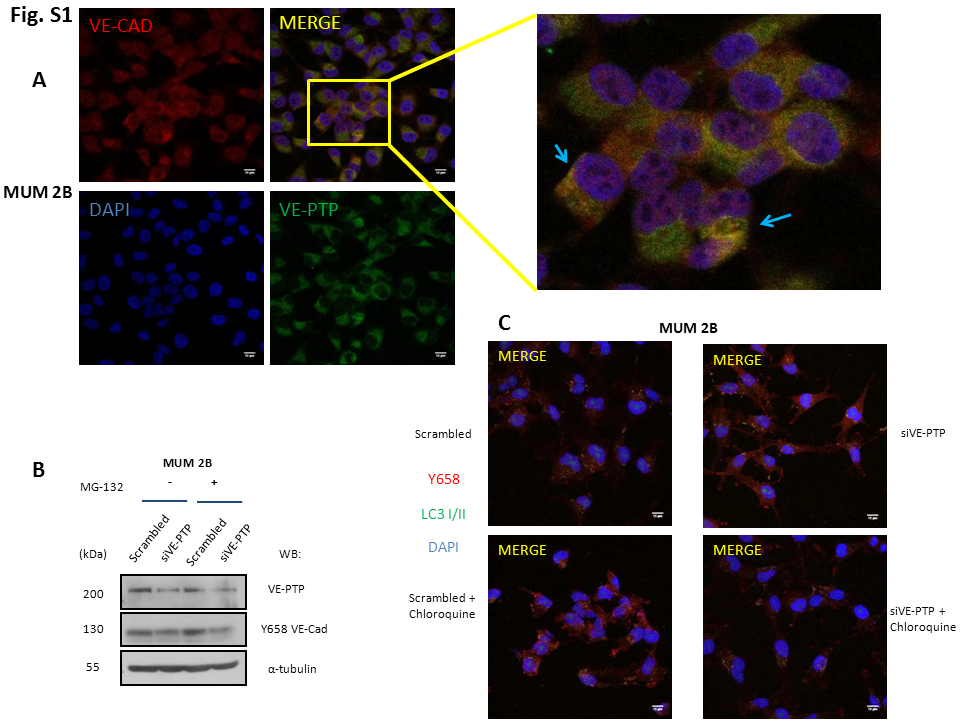

Supplement: Figure S1 — (A) Co-immunofluorescence of VE-PTP (green) and VE-Cadherin (red), DAPI (nuclear stain, blue) in MUM 2B cells. Bars 15 μm. (B) Inhibition of proteasome through MG-132 (3 μM during 3 h) with or without siVE-PTP conditions not prevent the VE-cadherin degradation. (C) Co-immunofluorescence of Y658 VE-Cadherin (red) and LC3 I/II (green) with or without siVE-PTP (Cloroquine treatment: 20 μM during 3 h) conditions in MUM 2B cells. Bars 15 μm. [file Image_1.tif]

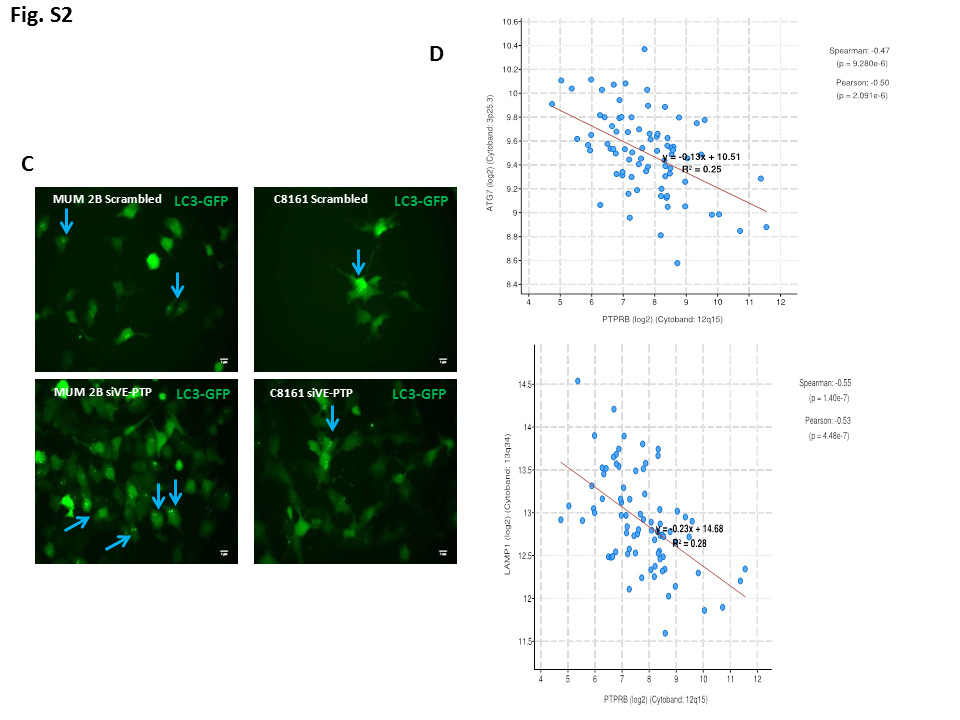

Supplement: Figure S2 — (A) Quantification of autophagosomes (LC3-GFP punctuated cells) in MUM 2B cells and C8161 cells after LC3-GFP transfection (0.5 μgr). (B) cBioPortal database, a platform of 48,333 tumors samples, we found that high mRNA levels of PTPRB, associated with the expression of two essential autophagy genes, ATG7 and LAMP1 in uveal melanoma samples. [file Image_2.tif]
